# Expression of soluble, active, fluorescently tagged hephaestin in COS and CHO cell lines

**DOI:** 10.3906/biy-2005-39

**Published:** 2020-12-14

**Authors:** Elif Sibel ASLAN, Kenneth N. WHITE, Basharut A. SYED, Kaila S. SRAI, Robert W. EVANS

**Affiliations:** 1 Department of Molecular Biology and Genetics, Faculty of Engineer and Natural Science, Biruni University, İstanbul Turkey; 2 School of Human Sciences, London Metropolitan University, London UK; 3 Rightmetrics Ltd., New York, NY USA; 4 Division of Biosciences, University College London, London UK; 5 Metalloprotein Research Group, Division of Biosciences, School of Health Sciences and Social Care, Brunel University, Uxbridge UK

**Keywords:** Hephaestin, ceruloplasmin, ferroxidase, iron

## Abstract

Hephaestin (Hp) is a trans-membrane protein, which plays a critical role in intestinal iron absorption. Hp was originally identified as the gene responsible for the phenotype of sex-linked anaemia in the *sla* mouse. The mutation in the *sla* protein causes accumulation of dietary iron in duodenal cells, causing severe microcytic hypochromic anaemia. Although mucosal uptake of dietary iron is normal, export from the duodenum is inhibited. Hp is homologous to ceruloplasmin (Cp), a member of the family of multi copper ferroxidases (MCFs) and possesses ferroxidase activity that facilitates iron release from the duodenum and load onto the serum iron transport protein transferrin. In the present study, attempts were made to produce biologically active recombinant mouse hephaestin as a secretory form tagged with green fluorescent protein (GFP), Hpsec-GFP. Plasmid expressing Hpsec-GFP was constructed and transfected into COS and CHO cells. The GFP aided the monitoring expression in real time to select the best conditions to maximise expression and provided a tag for purifying and analysing Hpsec-GFP. The protein had detectable oxidase activity as shown by in-gel and solution-based assays. The methods described here can provide the basis for further work to probe the interaction of hephaestin with other proteins using complementary fluorescent tags on target proteins that would facilitate the fluorescence resonance energy transfer measurements, for example with transferrin or colocalisation studies, and help to discover more about hephaestin works at the molecular level.

## 1. Introduction

Iron is an essential micronutrient required by every human cell and has the capacity to accept and donate electrons readily by interconverting between ferrous, depicted as Fe(II), and ferric, depicted as Fe(III), forms. Hence, iron is important for oxygen transport, energy transfer, and nucleic acid biosynthesis. A normal adult man has 40–50 mg iron/kg body weight (Andrews, 1999; Wessling-Resnick, 2000). Although only about 0.1%–0.2% of iron (~7mg) is bound to transferrin (Tf) in extracellular fluids for transit, the turnover of this transported pool is highly dynamic, reaching 30–35 mg/day (Hirota, 2018; Sherwood et al., 2018; Wessling-Resnick, 2000).

The molecular basis of dietary iron absorption into the body is currently quite well understood. Dietary iron is absorbed mainly in the duodenum, either as ferrous iron transported across the apical membrane of the enterocyte via divalent metal transporter-1 (DMT1), or as heme iron transported possibly via the heme-responsive gene HRG (Knutson, 2017) or HCP1 (Lawen and Lane, 2013). Once inside the enterocyte, the iron can be stored or exported across the enterocyte basolateral membrane by the ferrous iron exporter ferroportin (FPN) (Knutson, 2017). Hephaestin (Hp) increases the efficiency of this process by oxidising the transported iron to its ferric form and promoting its release from FPN (Crichton, 2009; Han, 2011; Lawen and Lane, 2013; Deshpande et al., 2017), from which it is rapidly bound to *apo* -Tf for transport in the circulation to tissues (Lawen and Lane, 2103; Knutson, 2017). In short, Hp plays a role in the uptake of iron from the diet, whereas Cp helps with the redistribution of iron to the cells and tissues; however, how Hp interacts with Tf, Cp, and FPN is still unclear.

To understand this mechanism, iron metabolism-disordered animals, e.g., knockout (KO) mice and *sla* mice, have been studied as useful models of iron homeostasis. By studying *sla* mice, Hp was identified as a key component of intestinal iron transport. *Sla* mice develop moderate to severe microcytic hypochromic anaemia due to a block in intestinal iron transport—dietary iron is absorbed into the enterocyte, but export into the circulation is compromised. Hp has 50% sequence similarity with Cp, including the conservation of multiple copper binding sites (Vulpe et al., 1999; Griffiths et al., 2005) and is highly expressed in *sla* mouse intestine. Interestingly, in *sla* mice, this gene shows an in-frame deletion of 194 amino acids, probably resulting in a non-functional product (Ranganathan et al., 2012a; Deshpande et al., 2017). The researchers conclude that this protein is a transmembrane-bound ferroxidase that plays an important role in the iron export mechanism involving FPN and Tf (Hudson et al., 2008; Vashchenko and Macgillivray, 2012; Lee et al., 2012; Chen, 2018; Zheng et al., 2018). The identification of mutant hephaestin as the gene responsible for the sex-linked anaemia in *sla* mice provides an important link between iron and copper metabolism in mammals (Lieu et al., 2001).

However, recent investigations performed using intestinal Hp knockout indicate that the link between Hp activity and iron absorption is not simple (Doguer et al., 2017).

In the current study, we have investigated the recombinant Hpsec-GFP plasmid prior to transfection in the COS cell system to study the expression of bioactive, recombinant soluble hephaestin in COS and CHO cells and then to assess its ferroxidase activity and iron efflux ability.

In the current study, the main aim was to understand and investigate the role of Hp systemic iron homeostasis by studying the activity assays and expression studies.

Therefore, microscopy was used to study the intracellular distribution of Hp and ascertain whether the correct protein is expressed. The data for the recombinant GFP-tagged Hp plasmids showed different patterns of expression. Full-length Hp-GFP was also constructed to confirm the localisation of Hp for further analysis, but the data are not shown here.

## 2. Materials and methods

### 2.1. Construction of soluble hephaestin protein

**Figure 1:**
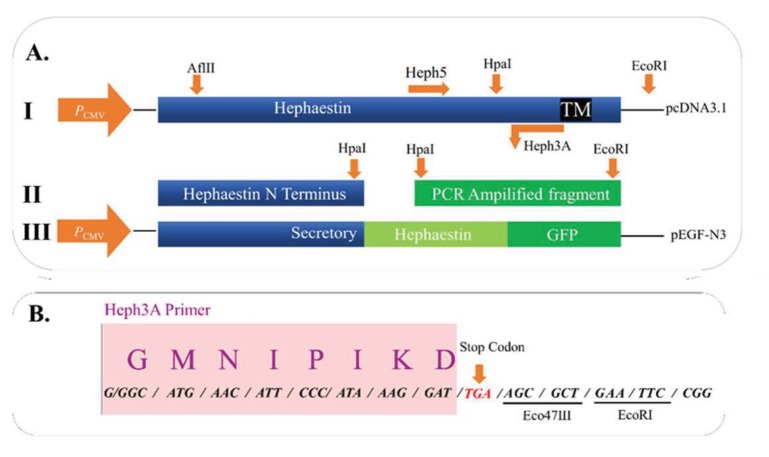
Plasmid construction for GFP-tagged secretory hephaestin. (A) The secretory form of hephaestin (Hpsec-GFP) was constructed for expression studies. (i) The full-length hephaestin cDNA was used as a template to amplify the 622 nt C-terminus fragment immediately upstream of the transmembrane I domain. (ii) The PCR product was religated into the pcDNA3.1 vector containing the N-terminal portion of hephaestin. Selected recombinants were sequenced through the full-length of the PCR amplicon. (iii) The recombinant hephaestin was fused to the N terminus of EGFP coding sequence. (B) The 3´ PCR primer was designed to include the Eco47III, EcoRI sites for the ease of subcloning and a stop codon. The single letter amino acid code is shown above the codons. The boxed region corresponds to the hephaestin sequence.

The Hp cDNA from mouse was subcloned from pcDNA3.1 into pEGFP-N3 as an expression vector for the expression of GFP-tagged proteins in eukaryotic cells to create GFP-tagged soluble and full-length forms of hephaestin (2 separate subcloning works, full length and truncated). For the soluble/secretory form of Hp devoid of the transmembrane domain (TM), a C-terminus fragment upstream of the TM domain was amplified using the primers Heph5 ( *5’-TCTGCAGAAATGGTATCTTGGAACC-3’* ) and Heph3A( *5’-CCGAATTCAAGCGCTATCCTTTATGGGAATGTTCATGCCC-3’* ), including a stop codon introduced just prior to the TM domain (Figure 1).

### 2.2. PCR amplification

A 50 µL reaction was prepared by mixing on ice 200 nM of each dNTP, 50 ng of template DNA, and 0.4 µM of each primer in 1xVentRbuffer [20 mM Tris-HCl (pH 8.8), 10 mM KCl, 10 mM (NH4)2SO4, 2 mM MgSO4, 0.1% Triton X-100]. Fifty µL of mineral oil were layered on top, and the reaction was subjected to a hot start treatment, i.e. the above reaction mix was incubated for 3–5 minutes at 94 °C before the addition of 2 U of VentR polymerase (New England Biolabs, Ipswich, MA, USA). Thermal cycling parameters were as follows: DNA denaturation at 94 °C for 1 min; primer annealing for 1 min at a temperature range of 55–72 °C, which was determined empirically for each template/primer pair; and primer extension at 72 °C, allowing 1 min per kilobase of expected extension product. A total of 30 cycles were carried out, followed by the incubation of the reaction at 72 °C for 10 min.

The PCR fragment was digested with HpaI and EcoRI restriction enzymes and gel purified prior to subcloning into the pcDNA3.1-Heph via HpaI and EcoRI sites (Figure 1) to create a cDNA, encoding a soluble/secretoryform of Hp (Hpsec). The truncated Hp construct was then subcloned into the Eco47III site of the pEGFP-N3 vector via AflII (blunted) and Eco47III to generate a cDNA, which expresses the recombinant Hpsec-GFP. The recombinants with correct orientation were selected by digestion with XhoI and the mutations were verified by sequencing.

### 2.3. Cloning and expression of the Hp protein

The bacterial cells INVαF’, JM109, and MC1061/P3, and the cloning vectors pcDNAI and pcDNA3.1 were purchased from Invitrogen (Paisley, UK). The pEGFPN3 was purchased from Clontech (Nottinghamshire, UK). The pcDNA3.1 is a high level constitutive mammalian expression vector. It has the hCMV promotor, which is able to drive high levels of transcription in a wide variety of mammalian cells (Foecking and Hofstetter, 1986). The NovaBlue *E. coli* K12 strain and the pSTBlue-1 vector were purchased from Novagen (Nottingham, UK). The pSTBlue-1 is also a multipurpose bacterial cloning and expression vector with versatile MCS, T7, and SP6 promoters and ampicillin and kanamycin resistance genes.

### 2.4. The COS cell system and passaging of cells

The COS-7 cells were obtained from the European Collection of Animal Cell Cultures. The cells were cultured in 9 cm disposable culture dishes in a moist atmosphere of 95% air and 5% CO2 at 37 °C. The COS cells were subcultured before confluency in the log phase of growth. The spent medium was discarded, and the monolayer of the cells was dislodged from the plate with a solution of 1x trypsin at 37 °C for 10 min. The cells were collected, sedimented, and the cell pellet was resuspended in complete RPMI 1640. Fresh plates were seeded with 1/10 volume of cell suspension and topped up with 9 mL of fresh medium.

### 2.5. Transfection using DEAE-Dextran

A day before the transfection, 3–5 x 105 cells per 9-cm culture dish were seeded. For each 9-cm dish, a transfection solution was prepared comprising 4.0 mL serum-free RPMI 1640, containing 200 mg/mL DEAE-Dextran and 10 mg of plasmid DNA, and the pH was adjusted to 7.4 with 0.4 mL 0.5 M Tris-HCl. After 24 h, the cells were washed 3 times with warmed serum-free RPMI 1640, and 4 mL of DEAE-Dextran-DNA mixture was added to each dish. The cells were incubated at 37 °C for 4 h. They were washed twice with serum-free RPMI 1640 and were then exposed to a RPMI 1640 7% FCS (v/v) solution containing 100 mM chloroquine and incubated at 37 °C for 3 h. Finally, the cells were washed twice, and cultured in full RPMI 1640 for 48–72 h.

### 2.6. Transfection of mammalian cells by electroporation

The electroporation of the COS and CHO cells was carried out using the preset protocol (160 V, 500 mF, exponential decay) in the Gene Pulser Xcell electroporation system (BioRad Laboratories, Inc., Philadelphia, PA, USA) experimentally with the optimum results obtained. The cells were pulsed once, immediately transferred to a culture dish, and cultured. Transient gene expression was assessed 24–48 h following electroporation with a fluorescent inverted microscope (Olympus 1X51, Olympus UK Ltd, Middlesex, UK). The transfected cells were usually fixed in 2% (w/v) paraformaldehyde in PBS for 1 h at room temperature prior to observing GFP fluorescence. One day after plating on coverslips, the GFP-tagged Hp constructs were transfected into COS-7 and CHO. Then, the cells were fixed in 2% (v/v) formaldehyde in PBS for 10 min. The images were then collected using confocal microscopy (Carl Zeiss Microscopy GmbH, Oberkochen, Germany).

### 2.7. Measurement of the hephaestin and Cp protein oxidase activity assay

A) 0.1% o-dianisidine oxidase assay

The samples obtained from the cell culture were subjected to PAGE (10% gel) under native conditions (nondenaturing). After electrophoresis, the gel was briefly rinsed in 0.1 M sodium phosphate buffer with ph 5.7. The oxidase activity was assayed qualitatively by incubating with the same buffer containing 0.1% o-dianisidine (3, 3´-Dimethoxybenzidine) and 30% ethanol at 37 °C for 60 min. The gel was left at room temperature until the staining for oxidase activity was clearly visible.

**B** ) p- **phenylenediamine oxidase activity measurement**

Serum ceruloplasmin *p* -phenylenediamine (PPD) oxidase activity was measured. At ph 5.6, ceruloplasmin catalysed the oxidation of ppd to yield a coloured product. The 0.45 M acetate buffer solution with ph 5.8 was prepared as follows: 36.91 g of CH3COONa was dissolved in 1000 mL of deionized water. Reagent grade glacial acetic acid (25.87 mL) was diluted to 1000 mL with deionized water (final concentration, 0.45 M). The sodium acetate solution (940 mL) was mixed with 60 mL of acetic acid solution under a ph meter; the ph of the acetic acid-sodium acetate buffer was 5.8. Then, 0.1 g *p-* phenylenediamine substrate was dissolved in sodium acetate buffer, treated with Chelex-100 by mixing by a rotator for 15–20 min, and centrifuged for 3–5 min at 700 rpm to collect the Chelex pellets to avoid interference in the assay. The buffer and sample mixtures were warmed at 37 ° C before the substrate was added at 1 min intervals. After this step, 10 μL (or 100 μL) aliquots of the sample in lysis buffer were assayed in a total volume of 1.0 mL (or 10 mL). The absorbance of each sample at 530 nm was measured using the CECIL 1021 Spectrophotometer at certain time intervals.

### 2.8. Western blotting

The proteins from SDS-PAGE were transferred to HybondTM-C extra membrane and optimised for protein transfer from Biosciences UK Ltd. (Amersham, UK) using a wet Western blot system (Hoeffer) run at 100 ma for 1.5 h or 15 ma overnight. Following blocking with 1% (w/v) skimmed milk powder (Marvel) in 0.05% (v/v) Tween-20, TBS, the nitrocellulose was then incubated for 2 h with either rabbit antimouse hephaestin (Alpha Diagnostic International, Inc., San Antonio, TX, USA) or anti-GFP antibody (Abcam Ltd., Cambridge, UK). All antisera were diluted to 1:500 in TBS prior to use. The sheet was washed 3 × 5min with TBS [0.05% (v/v) Tween-20]. Following the secondary antibody incubation, the membrane was incubated with 4 μL of streptavidin-alkaline phosphatase in 3 mL of 1% blocking buffer for 1 h in constant shaking at room temperature. The membrane was then incubated with freshly prepared substrate solution [60 μL of nitro-blue tetrazolium (NBT) (Sigma-Aldrich Corp., St. Louis, MO, USA), 60 μL of 5-bromo-4-chloro-3-indolyl phosphate (BCIP), and 5 mL of alkaline phosphatase buffer]. After the overnight incubation in darkness for band development, the membrane was washed with distilled water to quench the developing reaction.

### 2.9. Immunoprecipitation of recombinant hephaestin

Rabbit polyclonal anti-GFP antibody with a volume of 0.5 µL was added to 10 mL of cell medium (or lysate) containing recombinant Hp and was incubated on ice for 1 h. Then, 50 µL washed Pansorbin (10% (v/v) in lysis buffer: 0.5% TX-100 in PBS) was added to the mixture and further incubated for 1 h at 4 °C on a rocking platform. The beads were pelleted by centrifugation at 10,000 g for 15 s at 4 °C, the supernatant was removed by gentle aspiration, and the immune complex was washed on the beads 3 times with lysis buffer. Each time, 1 ml of lysis buffer was added and the beads were resuspended by pipetting. Following this, 50 µL of 1 × SDS gel-loading buffer was added to the washed pellet and the proteins were denatured by putting the tube in boiling water for 5 min. The sample was centrifuged for 15 s at 10,000 g and the supernatant was loaded on the gel.

## 3. Results

### 3.1. Expression of the recombinant Hpsec-GFP in the COS cells

**Figure 2:**
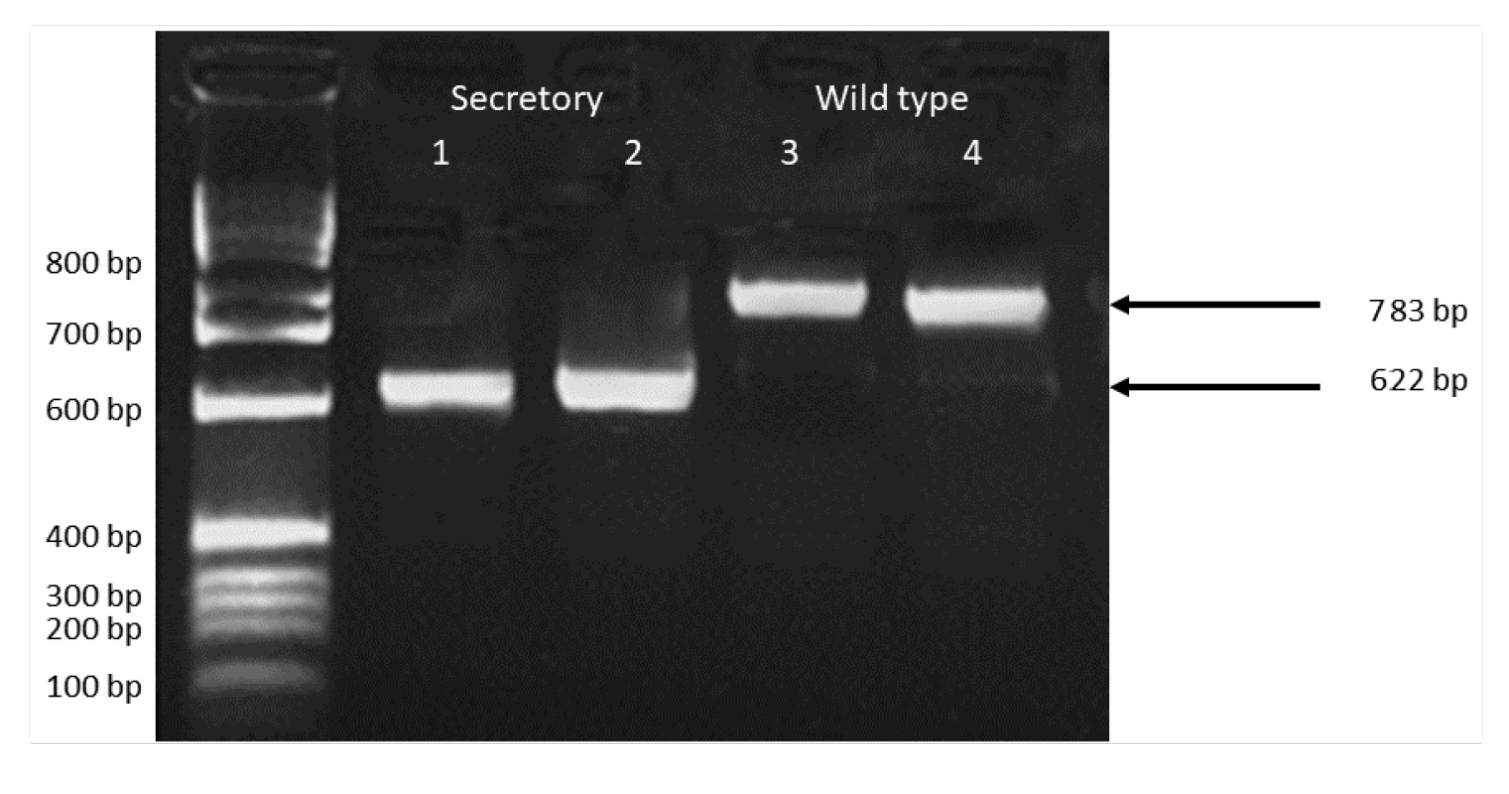
PCR products for generation of recombinant Hp constructs. 10 μL samples of PCR products were electrophoresed on a 1% agarose gel for 1 h at 100 mA. Lanes 1 and 2 contain the amplicons of the C-terminus of the secretory hephaestin construct produced using primers Heph5 and Heph3A described in Methods 2.1. Lane (M) is the 100 bp ladder (New England Biolabs). Lanes 3 and 4 contain amplicons of the C-terminus of the full-length (wild type) Hp with stop codon ablated.

**Figure 3:**
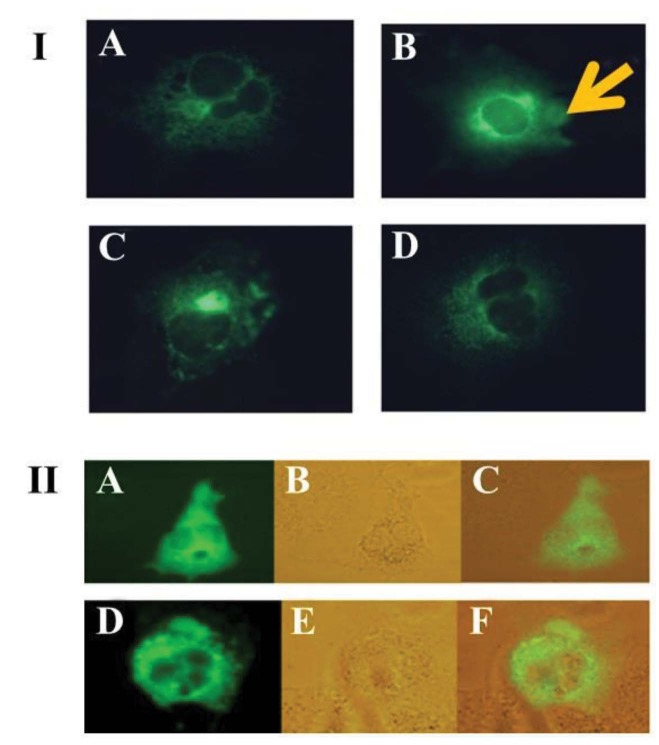
Expression of Hpsec-GFP in COS cells. (i) A strong green fluorescent signal was detected in the perinuclear region as indicated by the arrow in (B) with punctate signals throughout the cytoplasm, gradually decreasing in intensity, moving from the nucleus to the plasma membrane in the A, B, C, and D. (ii) Fluorescent signals (A and D) were checked by phase contrast shown in the middle panels (B and E); partial signal in C and F. Cells were transiently transfected using the DEAE-dextran method.

**Figure 4:**
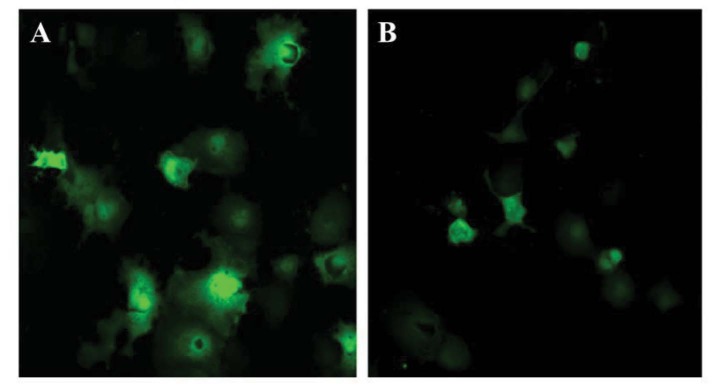
Expression pattern of GFP protein. GFP shows a distinct pattern in which staining is evident throughout the cell, inducing the nucleus and therefore are easy to compare with Hp- GFP constructs showing in A and B. Cells were transiently transfected with pEGFP-N3 using the DEAE-dextran method.

GFP-tagged soluble Hp was prepared for expression studies (Figures 1 and 2). The Hpsec-GFP construct was initially tested in a COS cell system. The COS cells were chosen for transfection because of their convenience to generate stable cell lines. Moreover, they provide a high level of expression and are adherent cells and therefore quite appropriate for microscopy studies. The intracellular distribution of Hp was checked by fluorescence microscopy and the expression profile of Hp was compared with the control GFP expression system, which has a distinct expression pattern (Figures 3 and 4).

Hpsec-GFP was transfected into the COS-7 cells using DEAE-Dextran or lipofectamine TM reagents. Figure 3 shows the expression of these recombinant Hp molecules, which is the expected expression pattern for the soluble Hpsec-GFP construct. The pattern correlates well for a secretory protein localisation with a strong perinuclear expression, and a vesicular pattern of localisation throughout the cytoplasm, suggesting that the protein is likely to be secreted. Moreover, the expression profile was distinct from GFP control construct (Figure 4), in which staining was uniform throughout the cell, including the nucleus. Taken together, the patterns of localisation suggest that the construct is expressing chimeric Hpsec-GFP with the patterns of localisation broadly expected for soluble protein. However, further experiments were needed to confirm that the correct protein was being expressed. Thus, the expression was further confirmed by Western blotting and biological activity was assessed by monitoring the oxidase activity.

### 3.2. Immunoprecipitation (IP) method for the preparation of GFP-tagged soluble hephaestin and the requirement of heating for elution

**Figure 5:**
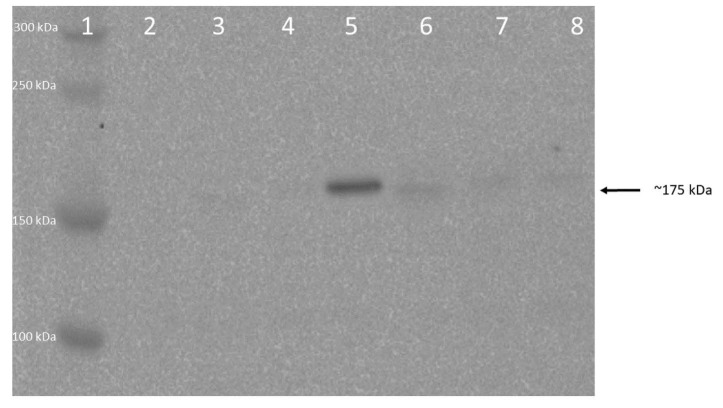
Identification of the recombinant Hpsec-GFP protein from CHO cells by Western blot. The cell culture medium or extracts were immunoprecipitated with rabbit anti-GFP antibody, incubated with Pansorbin and detected with the same antibody. The protein samples were electrophoresed on a 10% gel blotted and analysed with anti-GFP antibody and the figure shows immunoprecipitated samples prepared with heating at 90 °C for 5 min vs. those prepared without heating. Lane 1 contains broad range protein standard (New England Biolabs); lane 2 represents a negative control, immunoprecipitation of medium; lanes 3 shows a negative control, immunoprecipitated medium from untransfected CHO cells; lanes 4 and 5 are Hpsec-GFP prepared from transfected medium which gave a correct band for the sample eluted by heating (lane 5) at the end of precipitation, around 175 kDa indicated by arrow, but only a faint band for the sample prepared from the same medium but without heating (lane 4). Lanes 6 and 7 show immunoprecipitates prepared Hpsec-GFP cell extract, no band for the unheated samples of soluble hephaestin; lane 8 represents positive control, transfected IRP1-GFP which is around 125 kDa. The figure shows that the heating was necessary to elute the protein.

In this study, the recombinant soluble Hp protein was analysed from the cell culture medium from transfected CHO cells by using an anti-GFP antibody. The immunoprecipitation technique was used to increase the yield of proteins for the analysis. The expected size of the protein was 150 kDa for Hpsec-GFP, based on the size of the Hpsec and GFP polypeptides (Syed et al.,2002). Specific bands were successfully detected from the immunoprecipitated sample heated at the elution step, with molecular weight of 175 kDa (Hpsec-GFP) (Figure 5, lane 5) on Western blot analysis. Hpsec-GFP was expressed in the CHO cells, which were cultured in the selection medium containing G418. The CHO cells expressing Hpsec-GFP took around 14–16 days to reach t=70%–80% confluency. During the culturing, the Hpsec-GFP expression was closely observed by fluorescent microscope because the collection time of the cell culture media was critical for the preparation of the sample. Therefore, cell culture studies were strictly and carefully followed. The cell culture media were collected from 3 plates, making the total volume between 30–50 mL (9-cm dish), and then the immunoprecipitation technique was carried out. An important part of the technique was the elution step, which was performed by heating the Pansorbin carrying the immunoprecipitates at 90 °C for 4–5 min. Although heating elution was potentially very risky for the protein, the Hpsec-GFP prepared by heating yielded effective results.

**Figure 6:**
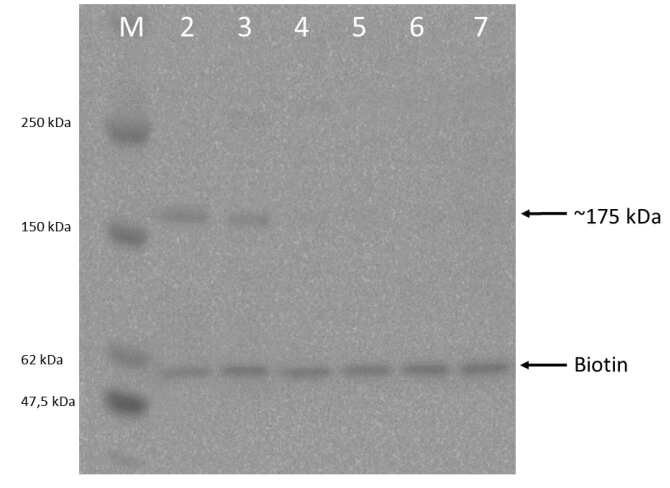
Western blot analysis of the hephaestin-GFPs expressed from COS cells. The cell culture medium or cell extracts were immunoprecipitated with rabbit anti-GFP antibody, incubated with Pansorbin and detected with the same antibody. The protein samples were electophoresed on a 10% SDS gel. Lane M contains broad range protein standards (New England Biolabs); lanes 2 and 3 are immunoprecipitates of medium from cells, transfected with pHpsec-GFP treated without and with Cu2+ respectively, eluted with heating; lane 4 represents a negative control, the immunoprecipitate of untransfected COS cell medium; lane 5 represents cellular extracts of untransfected COS cells (negative control); lanes 6 and 7 are extracts of Hpsec-GFP transfected cells treated without and with Cu2+ respectively. Recombinant soluble hephaestin could be detected in immunoprecipitates of cell culture medium, but not of cell lysates.

Specific bands were also successfully detected from immunoprecipitates prepared from the transfected COS cells, also with a molecular weight of 175 kDa (Hpsec-GFP) (Figure 6). The expected size of the recombinant polypeptide for the Hpsec-GFP polypeptide was 150 kDa (Syed et al., 2002). The difference between the predicted polypeptide size and the actual size of the recombinant product could be due to glycosylation, which will be further confirmed. Once again, the bands were observed when immunoprecipitates were eluted with heating (Figure 6, lanes 2 and 3). One set of COS cells were treated with 20 µM CuSO4 to see whether the yield of Hpsec-GFP would be increased (Figure 6, lane 3). However, there was no observable difference compared with Hpsec-GFP prepared from untreated cells (Figure 6, lane 2).

### 3.3. Oxidase activity of the recombinant hephaestin

**Figure 7:**
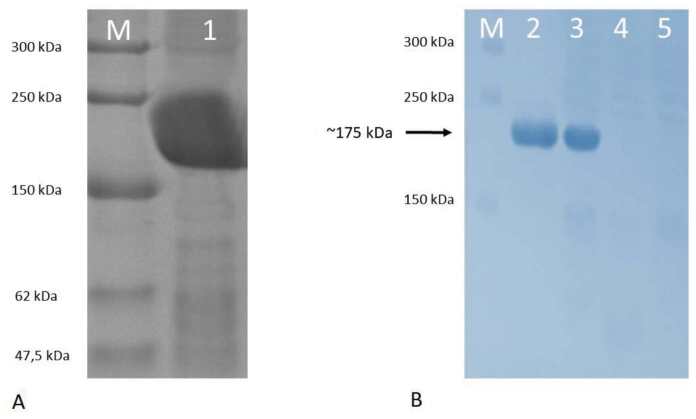
Coomassie staining and Oxidase activity of hephaestin expressed from CHO cells. 10% PAGE under nondenaturing conditions was used to separate the Hpsec-GFP expressed from CHO cells in the medium. Duplicates of Hpsec-GFP samples were loaded. After electrophoresis, the gel was cut in half and treated differently according to the protocols. Bands were identified with coomassie staining and oxidase assay on nondenaturing gel. (A) Oxidase staining was performed with o-dionisidine for the oxidase activity. Lane 1 contains an immunoprecipitated of Hpsec-GFP. (B) Coomassie staining was performed for 1 h in staining solution prior to overnight incubation in destaining solution; lanes 2 and 3 contain the secretory hephaestin purified by immunoprecipitation from transfected medium expressed in CHO cells; lanes 4 contain extract of CHO cells expressing full-length hephaestin and lanes 5 contains dialysed histidine tagged hephaestin. Western blot analysis confirms presence of single band of 175 kDa and oxidase activity was detected for the Hpsec-GFP sample from the same preparation (Figure 8).

**Figure 8:**
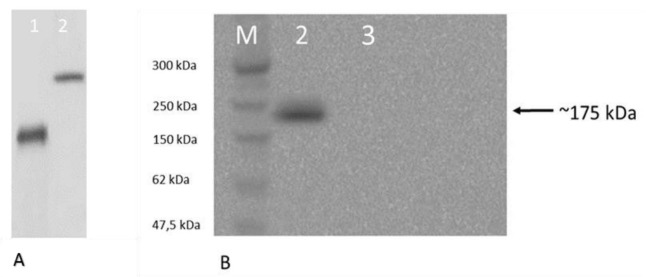
Gel oxidase assay and western blotting of hephaestin expressed from CHO cells. 10% PAGE under nondenaturing conditions was used to separate the Hpsec-GFP expressed from CHO cells in the medium. After electrophoresis, the gel was cut in half and treated differently for oxidase assay and Western blotting analysis for the same set of samples. Bands were confirmed to be hephaestin both with oxidase staining and by western blotting on native on nondenaturing gel. (A) Oxidase staining was performed with o-dionisidine for the oxidase activity. Lane 1 contains 5 μg of ceruloplasmin (Cp) as positive control; lane 2 represents the secretory hephaestin purified by immunoprecipitation from transfected medium expressed in CHO cells. (B) Western blot analysis of immunoprecipitated Hpsec-GFP expressed from CHO cells. Lane M contains broad range protein standards (New England Biolabs); lane 2 represents an immunoprecipitated pHpsec-GFP from CHO cells and lane 3 shows the immunoprecipitate of untransfected CHO cell medium. The arrows indicate the bands of Hpsec-GFP from either gel, which had the same mobility.

**Figure 9:**
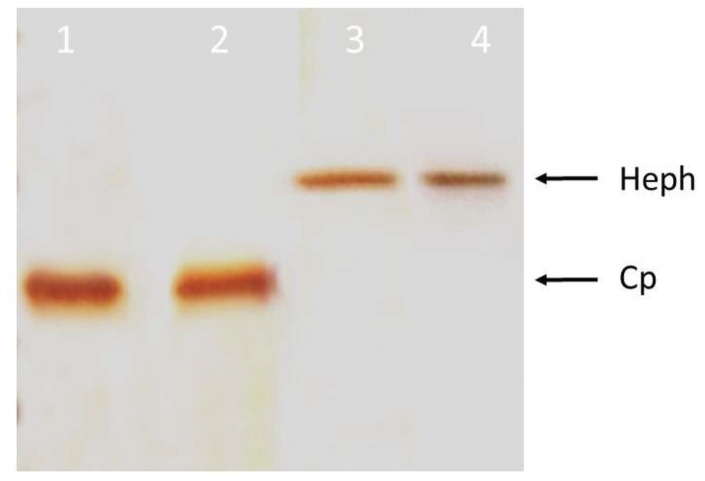
Oxidase activity of hephaestin expressed from COS cells. 10% PAGE under nondenaturing conditions was used to separate the Hpsec-GFP expressed from COS-7 cells in the medium. Lanes 1 and 2 contain 5 μg of ceruloplasmin (Cp) as positive control, and lanes 3 and 4 contain the secretory hephaestin purified by immunoprecipitation from transfected medium expressed in COS-7 cells. Staining was performed with o-dionisidine for the oxidase activity.

Having established that Hpsec-GFP can be successfully expressed, oxidase activity was assessed by the method of Johnson et al. (1967). Hpsec-GFP was purified from the medium using immunoprecipitation with a rabbit polyclonal antibody directed against GFP. The extracted protein was run on a native gel and oxidase activity was assayed using α-phenylenediamine/o- dianisidine substrate. Purified human Cp was used as a positive control. In the first experiments, the immunoprecipitates were compared for oxidase activity and protein content following electrophoresis of duplicate samples on the same gel (Figure 7). A single band of oxidase activity is evident (Figure 7A), which has the same mobility as a single band of protein identified by Coomassie staining (Figure 7B). The same preparations were analysed for oxidase activity (Figure 8A) and for Hpsec-GFP expression by Western blotting (Figure 8B). Cp was used to compare oxidase activity (Figure 8A, lane 1) in the nondenaturing gel and had a faster mobility than Hpsec-GFP (Figure 8A, lane 2). The Hpsec-GFP prep gave a single band of 175 kDa on Western blotting (Figure 8B). Similarly, the Hpsec-GFP expressed from the COS cells also had a measurable oxidase activity (Figure 9). Taken together, these data demonstrate that Hpsec-GFP has oxidase activity and that the recombinant protein has folded successfully even though it is a secretory form.

### 3.4. The measurement of solution-based oxidase activityor p-phenylenediamine oxidase activity

Having established that the recombinant Hpsec-GFP had oxidase activity, it was of interest to use a solution-based oxidase assay to obtain quantitative data. Stably transfected CHO cells were used to express Hpsec-GFP. The expressed Hpsec-GFP was obtained 14–16 days after the transfection and the expression level was checked by fluorescence microscopy 3 times a week. Each time, fresh medium containing G418 was added. When the Hpsec-GFP cells reached an expression level of 70%–80%,the cell medium was collected from 3 or more plates. The samples were prepared by immunoprecipitation from a total of 50 mL cell medium and the samples were eluted by heating at 90 °C for 4–5 min.

**Figure 10:**
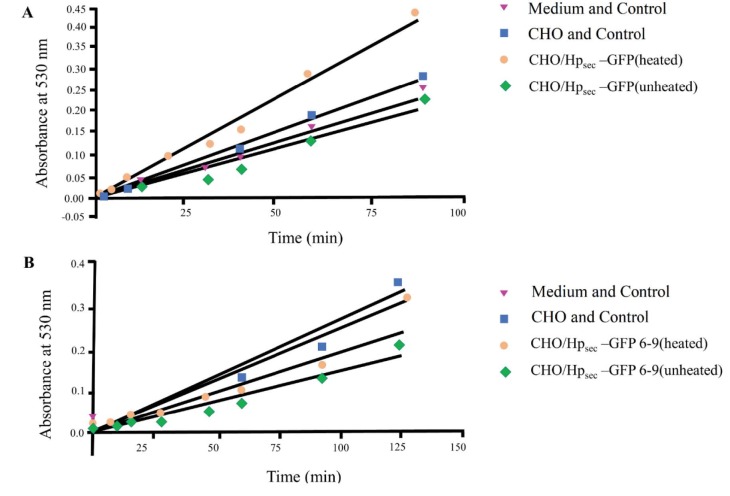
Oxidase assay of recombinant Hpsec-GFP for heated and unheated samples. (A) Oxidase activity of heated soluble hephaestin expressed in CHO cells. The active samples have about 30% activity above the negative controls. Immunoprecipitated protein eluted with heating on the final stage of preparation has more activity than unheated precipitates. As seen on the graph Hpsec-GFP (unheated), so heating is necessary to elute activity after immunoprecipitation. (B) Oxidase assay of recombinant Hpsec-GFP for heated and unheated samples after 10 days storage at 4 °C: Immunoprecipitated medium of Hpsec-GFP sample with heating on the final stage of protein elution are orange and Hpsec-GFP without heating are green which were assayed after 10 days; blues represent CHO and control; pink are the medium as negative control. Oxidase activity of the soluble hephaestin samples were assayed after 10 days showed less activity than freshly assayed ones (see Figures 10A and 10B). Data are the means of duplicates and are single experiments representative of three batches. Error bars are omitted for clarity and standard deviations were less than 15% of the mean.

Oxidase activity was assayed by the method of Sato and Gitlin (1991). The Hpsec-GFP oxidase activity was assayed using α-phenylenediamine substrate. Protein was assayed by the Bradford method and the equivalent amounts were assayed per experiment, typically about 5 µg protein. Oxidase activity could be detected in immunoprecipitates from the medium of cells expressing Hpsec-GFP. Figure 10 shows data from one set of assays.

In this experiment, the effect of elution by heating was analysed by preparing the immunoprecipitates with and without heating on the final stage of protein elution. As seen on the graph (Figure 10A), heating is necessary to elute activity after immunoprecipitation. The samples were stored at 4 ºC for 10 days and reassayed. Figure 10B shows that oxidase activity was lost over this period, and that freshly prepared samples had to be used shortly after preparation for oxidase studies.

### 3.5. The effect of copper on the expression and activity of the hephaestin protein

The availability of copper in the system may also contribute to the levels of expressed hephaestin. In order to address this concern, 20 µM Cu2+was supplemented to the medium to try to increase the yield of proteins. Further addition of copper did not seem to increase the levels of expression. The possible reason could be the toxic effect of copper on cells because the survival times of cells treated with Cu2+were shorter than cells without extra Cu2+.

**Figure 11:**
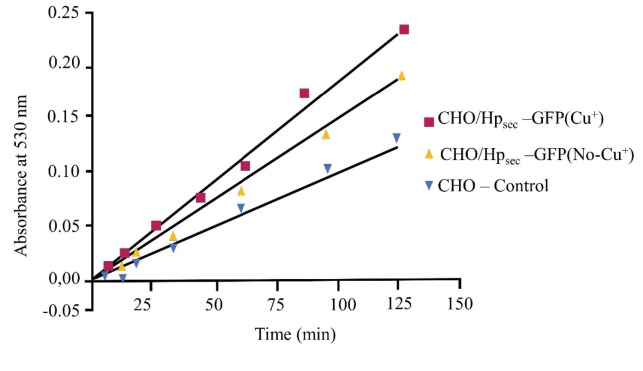
Oxidase assay showing activity of soluble hephaestin expressed from cells with and without copper treatment. Oxidase activity of soluble hephaestin expressed in CHO cells treated with/out Cu+. Cells treated with 20 μM copper are in dark red; cells without copper treatment are orange. Cu+ treated cells showed more activity than cells without Cu+ treatment, but there were no significant differences on repeated assays (data not shown here). Data are the means of duplicates and are single experiments representative of three batches. Error bars are omitted for clarity and standard deviations were less than 15% of the mean.

In this experiment, the cells were treated with/out copper to investigate the requirement of copper for oxidase activity. Stably transfected CHO cells expressing Hpsec-GFP had a slight increase in oxidase activity when treated with copper, compared with untreated samples, but on Western blots, no band was detected from either treated or untreated samples. Even though a slightly increased oxidase activity was found, the cells treated with copper were less viable and protein yields were low. Future work is needed to investigate the effect of copper of lower concentrations to find the optimal concentration to increase yield without affecting cell growth (Figure 11).

## 4. Discussion

In recent years, a number of key proteins involved in iron metabolism have been identified, although this information has not necessarily clarified certain aspects of cellular iron metabolism. Hp was identified as a key component of intestinal iron transport through the study of *sla* mice (Vulpe et al., 1999; Ranganathan et al., 2012a).

The expression construct for Hpsec-GFP (Figures 1 and 2) produces a protein which is analogous to Cp, a soluble serum protein produced in the liver. On microscopy, the secretory form of Hp gave a strong green fluorescent signal in the perinuclear region with punctuate signals in the cytoplasm, gradually decreasing in intensity, moving from the nucleus to the plasma membrane (Figures 3 and 4). The expression profile of Hp is distinct among the molecules involved the iron metabolism pathway. In our results, this profile is consistent with the expression expected for soluble proteins (Shen et al., 2005).

The COS cell and CHO expression systems used in this study produced usefully measurable amounts of the recombinant Hpsec-GFP protein, as was confirmed by Western blotting of immunoprecipitates from the transfected COS and CHO cell media (Figures 5 and 6). The expected size of protein was 150 kDa for Hpsec-GFP (Syed et al., 2002) and specific bands were successfully detected from immunoprecipitates with a molecular weight of 175 kDa according to Western blot analysis. Glycosylation was expected to account for the difference between the recombinant products with a predicted polypeptide size of 150 kDa and the actual size of 175 kDa.

In an attempt to confirm this expectation, the set of samples was treated with *Endo* H enzyme to remove the carbohydrate chains. However, this treatment did not result in a change in the product size, possibly indicating that sugar moiety was resistant to this enzyme.

Through the use of in-gel assays with an o-oxylamine or phenylenediamine substrate, the Hpsec-GFP protein was shown to possess oxidase activity (Figures 7–9). During the current study, several experiments showed that heating was necessary for the elution step of the immunoprecipitation; the samples without heating did not yield any protein and activity (Figures 5 and 10). Although the elution of a protein by heating is risky, the Hp samples prepared by heating did not denature. Attempts were made to assay the oxidase activity of the immunoprecipitates still bound to Pansorbin cells, but they were not accurate. As such, it was not possible to measure how much oxidase activity may have been lost by the heat treatment (Figure 10). Its ability to withstand heat treatment shows that Hp has a stable structure, but this may be because GFP helps stabilise the protein. These results indicate that the recombinant protein folds properly and has copper correctly incorporated into it in order for it to have biological activity.

Interestingly, Kuo et al. (2004) demonstrated the presence of detectable oxidase activity for the truncated protein in *sla* mice, suggesting the possibility that other factors may also contribute to the decreased iron exit in *sla* mice. Indeed, it was also reported that the Hp protein is mislocalised in *sla* enterocytes. It has been suggested that the Hp ferroxidase activity may be necessary for the effective release of iron from enterocytes, with or without FPN interaction, in order to transport iron across the basolateral membrane (Vulpe et al., 1999). The exact role of Hp in the efflux process therefore still remains unknown, but it is important to note that the possible sites for Hp action might be on the basolateral surface with the C-terminus in the cytoplasm, or in a vesicle (Syed et al., 2002; Ranganathan et al., 2012b).

As noted above, Hp is a copper-containing protein homologous to Cp, and its ferroxidase activity may aid the binding of iron to apotransferrin since only Fe(III) binds (Linder et al., 2003). Interestingly, according to Chen et al. (2006), copper is likely not necessary for the proper folding or assembly of most copper proteins, including the multicopper oxidases. Furthermore, it does not dramatically alter their subsequent stability (Chen et al., 2006). Genetic or nutritional copper deficiency does not change the levels of inactive *apo* -Cp, although the protein has a decreased plasma half-life. In contrast, the finding that copper deficiency decreased the Hp protein levels suggests an unexpected and unusual link between copper availability and protein stability (Figure 11).

The availability of copper in the system may contribute to the levels of expressed Hp. Further studies are needed to determine whether copper has a role in Hp expression and whether the protein level and oxidase activity are reflective of a copper-related process. To address this concern, an additional 20 µM Cu2+ was added to the medium to try to increase the yield of proteins in the system. This addition of copper did not increase the levels of expression. The possible reason is that the addition of a higher concentration of copper or a nonoptimal concentration of copper to the cells could exert toxic effects on cells. Although the copper-treated cells had some oxidase activity, no band was detected on Western blotting analysis; therefore, only this single dose was applied during this analysis (Figure 11).

According to the literature, intestinal iron transport pathways are also Cu sensitive. It has been suggested that copper deficiency chronically impairs Hp activity, which leads to inefficient basolateral intestinal iron export, as evidenced by increased enterocyte ferritin levels and systemic iron deficiency and anaemia. It was found that copper deficiency does not affect Hp transcript levels in HT29 cells but results in dramatic decreases in Hp protein and PPD oxidase activity. Together, these studies provide evidence that copper deficiency does not change mRNA levels but decreases Hp protein levels and subsequently oxidase activity. The mechanisms of copper loading into copper proteins in the secretory pathway are not known and need to be clarified (Chen et al., 2006).

## 5. Conclusion

In summary, we were able to produce a recombinant secretory form of Hp tagged with GFP. The GFP tag was helpful for monitoring the expression levels in real time, helping to optimize conditions for the maximal expression and also providing a tag for purifying and analysing the protein. We were able to demonstrate a measurable oxidase activity of the recombinant protein. We also demonstrated that heating the immunoprecipitates prepared using Pansorbin to 90 °C was necessary to free Hp for analysis. Despite the high temperature used, Hp had a demonstrable oxidase activity, suggesting that the recombinant protein was very stable. The methods described here provide a versatile means of producing recombinant Hp under conditions that can be easily achieved. In particular, the preparation of a secretory form of tagged fluorescent hephaestin may allow further studies to probe its interaction with transferrin, for example by fluorescence resonance energy transfer.
